# Improving Hand Hygiene Skills Using Virtual Reality: Quasi-Experimental Study

**DOI:** 10.2196/78882

**Published:** 2025-10-07

**Authors:** José Mira, Mery González, Carolina Villalba, Laura Guerra, Yesid Ramirez-Moya, Jazmín Hernández, Olga Moya, Luis Pineda, Clara Pérez-Esteve

**Affiliations:** 1 Fundación para el Fomento de la Investigación Sanitaria y Biomédica de la Comunitat Valenciana Alicante Spain; 2 Health Psychology Department Universidad Miguel Hernández Elche Spain; 3 Maestría de Gerencia en Seguridad y Salud en el Trabajo Corporación Universitaria Minuto de Dios Bogotá Colombia; 4 Oficina de Salud Pública y Epidemiología Clínicas Colsanitas Bogotá Colombia; 5 Oficina de Calidad Clínica de Occidente Bogotá Colombia; 6 Praxisalud Institución IPS (Institución Prestadora de Servicios de Salud) Bogotá Colombia; 7 Red Iberoamericana de Conocimiento en Seguridad del Paciente Bogotá Colombia

**Keywords:** hand hygiene, virtual reality, training, infection control, caregivers, health care education

## Abstract

**Background:**

Hand hygiene is a critical strategy for preventing health care–associated infections (HAIs) and reducing health care costs. However, adherence remains low, particularly among health care assistants (HCAs) and informal caregivers (ICs), who often lack formal training. Virtual reality (VR) delivers standardized, immersive practice with active learning and real-time feedback. It has shown favorable effects on skill execution and acceptability in training paramedics and caregivers. To our knowledge, VR has not been systematically applied to train World Health Organization (WHO)–aligned hand hygiene techniques. Given its portability and suitability for brief, repeatable drills, VR is a plausible solution to upskill HCAs and ICs in both hospital and home-care settings.

**Objective:**

This study aims to assess the immediate training effectiveness and implementation feasibility of a brief VR-based hand hygiene program for HCAs and ICs in Colombia. We quantified pre-post changes in correct execution (primary outcome), timing, errors, and knowledge. Success was defined a priori as achieving ≥75% correct execution after training, consistent with adherence levels associated with HAI reductions when embedded in WHO-aligned bundles in prior studies.

**Methods:**

In this quasi-experimental, one-group pretest-posttest study, 215 participants (94 HCAs, 121 ICs) completed up to three 15-minute VR training sessions with real-time feedback on hand hygiene technique following the WHO recommendations for hand hygiene. Data were collected at baseline (pre) and immediately after the VR intervention (post). Variables assessed included correct execution (primary; binary), error counts, timing adequacy, knowledge assessment, and acceptability.

**Results:**

Correct hand hygiene performance increased from 26.6% to 97.9% among HCAs (95% CI 92.6-99.4; *P<*.001) and from 9.9% to 95.9% among ICs (95% CI 90.7-98.2; *P<*.001), with paired odds ratios of 34.5 (95% CI 8.46-140.72) and 21.8 (95% CI 8.90-53.43), respectively. Wide intervals were driven by the very small number who performed worse after training. Timing adequacy improved significantly in both groups, reaching 46.6 (SD 6.7) and 48 (SD 6.6) seconds, respectively (*P<*.001). Common errors, such as insufficient fingertip coverage and incomplete thumb cleaning, were reduced to near 0 (*P*<.001). Knowledge scores also improved significantly in both groups, and VR training was rated as “very useful” or “extremely useful” for skill acquisition.

**Conclusions:**

VR training significantly improved hand hygiene technique and knowledge. The high acceptance rates observed suggest that these technologies can effectively enhance infection prevention skills in undertrained populations, supporting broader adoption in health care education. Because this brief, portable, and highly acceptable intervention can be embedded in routine onboarding, refresher microdrills, and caregiver education—including home care and resource-constrained settings—VR is well-suited for scale-up. When implemented within WHO-aligned multimodal bundles and with adherence sustained above pragmatic thresholds, this approach may contribute to downstream reductions in HAIs. Definitive confirmation will require a controlled effectiveness trial.

**Trial Registration:**

ClinicalTrials.gov NCT07005544; https://clinicaltrials.gov/study/NCT07005544

## Introduction

### Challenge

Hand hygiene is one of the most effective strategies for preventing nosocomial infections and reducing the morbidity and mortality associated with health care–associated infections (HAIs) [1]. In home settings, inadequate hand hygiene also contributes to the transmission of infections, leading to an avoidable health care burden that unnecessarily consumes resources [2]. Despite its critical role in infection prevention, adherence to proper hand hygiene remains low, ranging from 10% to 70% depending on the country [3,4]. In Colombia, nosocomial infection rates reach 2.55/1000 patient-days [5], leading to an increase in health care costs [6]. Hand hygiene compliance among health care professionals is estimated between 19% and 50% [7-9]; however, scarce data are available for the general population. Among schoolchildren, 34% reported washing their hands with soap and water before eating and after using the bathroom [10].

### Active Learning

Traditional hand hygiene training strategies have shown limitations in fostering the sustained acquisition of skills and attitude changes [11]. Active learning, combined with immersive experiences such as those provided by virtual reality (VR), has proven to be an effective approach for skill acquisition, knowledge retention, and attitude modification [12].

### VR Interventions

VR has been used to improve caregiving practices in dementia [13-15], cancer [16], and other care contexts [17] to strengthen resilience, particularly in coping with the characteristic burden experienced by caregivers [18,19], and to support the delivery of more complex care tasks [18], with favorable results, high acceptability, and effects sustained at least in the midterm. However, to our knowledge, it has not yet been applied to training a routine practice with persistent deficits—namely, hand hygiene.

### Hand Hygiene Skills

In hand hygiene, multicomponent bundles built around WHO’s Five Moments for Hand Hygiene framework typically produce absolute gains in adherence between 30 and 80 percentage points [20]. When postintervention adherence is sustained above roughly 75% and supported by system enablers, programs have been associated with meaningful reductions in HAIs [21]. Against this backdrop, VR-based training can serve as the formative component that standardizes technique and accelerates skill acquisition in both clinical and home care settings.

Health care assistants (HCAs) and informal caregivers (ICs) are key target groups for improving hand hygiene skills and awareness. Despite their essential role in patient care, these groups have historically received less attention in training programs, which predominantly focus on physicians and registered nurses [22]. ICs, in particular, are rarely included in structured hand hygiene training initiatives, despite their direct involvement in patient care at home. Given the increasing reliance on informal caregiving in low- and middle-income countries, targeted interventions for this group are urgently needed to reduce infection transmission and improve patient safety.

### Study Aim

This study aims to evaluate the immediate training effectiveness on correct hand hygiene execution and the implementation feasibility (acceptability, usability, and practicality) of a brief VR-based hand hygiene program for HCAs and ICs in Colombia. Immediate pre-post changes in correct execution (primary outcome), timing, and error counts were quantified. Knowledge was assessed as a secondary outcome, and feasibility was evaluated using acceptability, usability, and practicality measures. Training success was judged against a prespecified performance benchmark of a ≥75% correct execution after training, consistent with levels linked to HAI reduction in prior studies [21].

## Methods

### Study Design

This quasi-experimental study employed a one-group pre-posttest design, using paired data analysis. All participant data were anonymized using unique identifiers.

The description of the intervention followed the Template for Intervention Description and Replication (TIDieR) checklist [23] to ensure transparent and comprehensive reporting of the VR hand hygiene training (Multimedia Appendix 1). Given the nonrandomized design, we also followed the TREND (Transparent Reporting of Evaluations with Nonrandomized Designs) statement [24] for nonrandomized evaluations of interventions (Multimedia Appendix 2) and, where applicable, the CONSORT-EHEALTH (Consolidated Standards of Reporting Trials of Electronic and Mobile Health Applications and Online TeleHealth) checklist [25] for digital health interventions. The study was registered at ClinicalTrials.gov on March 6, 2025 (NCT07005544)

### Ethical Considerations

The study protocol was approved by the Research Ethics Committee of Unisanitas (CEIFUS 274-25) on January 29, 2025. The committee oversees several hospitals, including Colsanitas, in Bogotá, Colombia. All participants (HCAs and ICs) were adults and provided written informed consent prior to any study procedures. This manuscript reports primary data collected under the approved protocol; no secondary analyses beyond those specified in the protocol were undertaken. Study data were collected and stored in deidentified form, with no direct personal identifiers retained in the analytic data set. Records were stored on secure, access-controlled servers, and only the research team had access to them. The results were presented in aggregate to prevent reidentification. The participants did not receive any financial or material compensation. The manuscript and supplementary materials contain no images in which individual participants are identifiable. The study was conducted in accordance with the Declaration of Helsinki (latest revision) and applicable national regulations.

### Patient and Public Involvement

There was no patient or public involvement in the design, conduct, or analysis of the study.

### Study Period

The study was conducted between March and May 2025, during which no secular events occurred.

### Study Setting

This study was conducted in 7 private hospitals in Bogotá, Colombia.

### Outcomes

The primary outcome was correct hand hygiene assessed in the same participants immediately before and after training. Performance was evaluated by trained observers against guideline-based criteria aligned with WHO’s recommendations for hand hygiene. The correct execution was defined a priori as completing all 5 rubric steps without omissions or out-of-sequence actions (pass/fail).

Secondary outcomes included: (1) error counts; (2) hand hygiene knowledge, measured with a multi-item scale (higher scores indicate greater knowledge); (3) time spent performing hand hygiene; (4) self-reported confidence or perceived competence to perform hand hygiene in routine care; and (5) acceptability/usability of the VR training (brief postsession questionnaire).

### Eligibility Criteria

The eligibility criteria included facilities and services delivering care to a high volume of patients in need of home-based support, where ICs play an active role in daily care provision.

### Participants

A total of 140 HCAs from hospitals and 170 ICs of dependent patients in home settings were recruited. In the Colombian hospitals involved in this study, HCAs include all patient-facing health care support staff, such as auxiliaries, orderlies/porters, and similar roles with direct patient contact. Their activities are performed under the supervision of a registered nurse.

### Sample Size

The sample size was calculated to detect pre-post differences for the primary end point—correct hand hygiene performance of at least 15% among HCAs and 10% among ICs, with a 95% CI, 80% power, and an expected attrition rate of 15% and 20% respectively. This yielded a required sample of approximately 106 HCAs and 140 ICs in the postintervention phase. Secondary outcomes were prespecified as supportive and were not used to determine the sample size.

### Recruitment

The participants were selected using a snowball sampling technique among HCAs from 7 clinics in Bogotá and ICs accompanying hospitalized patients or those attending outpatient consultations. Participation was voluntary, and all participants provided informed consent. The inclusion criteria required participants to be 18 years or older and able to understand and follow instructions related to hand hygiene. HCAs had to be actively employed in a clinical setting with direct and regular patient contact, while ICs had to have been providing care to a dependent patient at home for at least the past 6 months. Caregivers with prior health care training or experience with VR were excluded. Participants with epilepsy or a history of seizures, as well as those with musculoskeletal or motor impairments that would prevent the performance of hand hygiene movements, were also excluded.

### Materials

**Figure 1:**
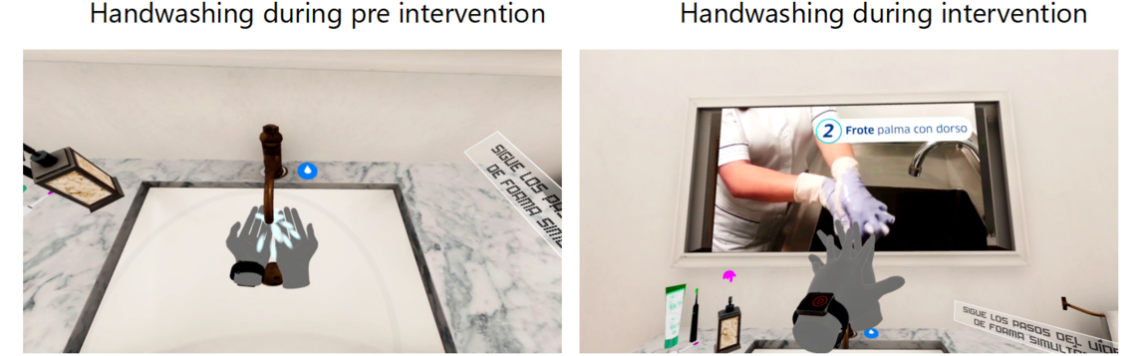
Virtual reality (VR) scenarios that simulated a complete handwashing sequence with water and soap.

This study utilized a VR scenario that simulated a complete handwashing sequence with water and soap, including an instructional display demonstrating the correct hand hygiene technique. During the intervention phase, participants performed handwashing as they would typically do, without any guidance. The intervention itself consisted of a standardized handwashing sequence supported by a 5-step instructional video lasting 1 minute and 27 seconds. For didactic purposes, the WHO-recommended handwashing technique was grouped into five labeled steps covering: (1) rubbing the palms together, (2) rubbing the backs of both hands, (3) interlacing the fingers to clean interdigital spaces, (4) cleaning fingertips and nails by rubbing against the opposite palm, and (5) rotational rubbing of both thumbs. Each movement was demonstrated at the recommended pace and accompanied by a short audio narration and concise on-screen prompts in imperative form. The virtual bathroom mirror served as an instructional display, demonstrating and indicating what to do and when to do it to ensure correct movements and timing. Prompts were displayed in Spanish to match the training context (Figure 1).

Training was delivered using Meta Quest 2 headsets (Meta Platforms). The training was conducted at each hospital in a dedicated, VR-ready room cleared of obstacles and supervised by a facilitator to minimize risks (tripping, falls, disorientation). Hygienic, wipeable headset covers were used, and the headset and controllers were disinfected between participants. Before starting, participants were screened for contraindications to VR headset use.

Knowledge was assessed using a 5-item multiple-choice test. The content was reviewed by a 3-member expert panel to ensure that each item accurately reflected current recommendations, resulting in item-level (I-CVI=1) and scale-level (S-CVI/Ave=1) content validity indices, indicating full agreement among experts on the appropriateness of the items. Higher scores on this 5-item knowledge test indicate greater knowledge.

A rubric was employed to assess accuracy and errors based on the standardized hand hygiene protocol approved by institutions involved in this study, which follows WHO recommendations. A total of 3 trained reviewers and 2 researchers assessed participant performance at both the pre- and postintervention periods. Consensus was established prior to the evaluations to ensure the reliability of the assessment. Two rounds of rater training were conducted using the rubric, with diverse worked examples (including borderline cases) to calibrate judgments and ensure reliable assessments. Additionally, a questionnaire assessed knowledge of proper hand hygiene, and 2 additional questions evaluated the acceptability of the intervention in improving hand hygiene compliance.

### VR Onboarding

Prior to the intervention, and to minimize motion-tracking issues and ensure smooth onboarding, participants completed a brief standardized orientation covering headset fit, basic navigation, and the gesture set required for the hand hygiene tasks.

### Intervention

The intervention was based on active learning principles [26]. It consisted of 4 stages. First, participants recorded their initial hand hygiene performance using water and soap, which served as the baseline assessment (pre). Next, they attended a preliminary session to familiarize themselves with the VR device. This was followed by up to three 15-minute training sessions in fully immersive VR scenarios, allowing for interactive hand hygiene practice with real-time feedback and conducting a posttraining evaluation. Each session covered key elements of proper hand hygiene, including step-by-step guidance on the correct sequence, duration, and coverage of handwashing movements. The participants received immediate visual and auditory feedback, highlighting mistakes and reinforcing correct techniques. At the end of each attempt, participants received immediate corrective feedback on an external monitor connected to the VR system. The display replayed the sequence just performed, while the facilitator highlighted missed areas, out-of-sequence actions, or insufficient duration (eg, fingertip coverage, thumb rotation, nail cleaning). The participants then reran the scenario to implement the corrections and reinforce learning. Each participant was allowed up to 3 attempts to improve their performance. For analysis purposes, only the last attempt was used to evaluate hand hygiene execution. The system also provided performance tracking to measure progress over time.

In the pre- and posttraining phases, participants completed a 5-question questionnaire assessing their knowledge of proper hand hygiene practices.

No concomitant treatment was provided that could interfere with the intervention. Participants received only the usual attention appropriate to their setting, with no additional interventions, support measures, or procedures introduced during the study period.

### Measures

This study assessed the instances of noncompliance with the correct hand hygiene protocol, types of errors in technique execution, time spent on hand hygiene, and correct responses to the WHO’s recommendations for hand hygiene. The time each participant spent washing their hands—both during the preintervention phase and the intervention—was recorded. The timer started when hand hygiene movements began and stopped when the water tap was closed.

Data collection was conducted by trained research assistants who administered the hand hygiene performance assessments and knowledge questionnaires in a controlled setting. Pre- and postintervention evaluations took place immediately before and after the VR training sessions.

### Blinding

The individuals responsible for administering the intervention recorded participants' hand hygiene performance using Quest 2 devices anonymously. All data were automatically and fully anonymized upon transfer to a secure database. The analysis was carried out by an independent researcher who was not involved in the implementation of the intervention, ensuring blinding during the data analysis phase.

### Data Analysis

We first assessed the missing-data mechanism with the Missing Completely at Random (MCAR) test by Little on key baseline and pre variables.

A linear mixed-effects model (LMM) with repeated measures was used to evaluate the intervention’s effects over time. Descriptive statistics and paired comparisons were performed to assess changes in hand hygiene performance. We compared paired proportions using the 2-sided McNemar test (α=0.05) and quantified change with the absolute difference in proportions (net increase in correct execution) and the McNemar conditional odds ratio with 95% CIs computed on the log scale. When a discordant cell was 0, a 0.5 continuity correction was applied. For continuous outcomes (timing), we used paired *t* tests; for ordinal totals (knowledge), we used Wilcoxon signed-rank tests; and for error frequencies, we used Fisher exact tests.

## Results

### Overview

**Figure 2:**
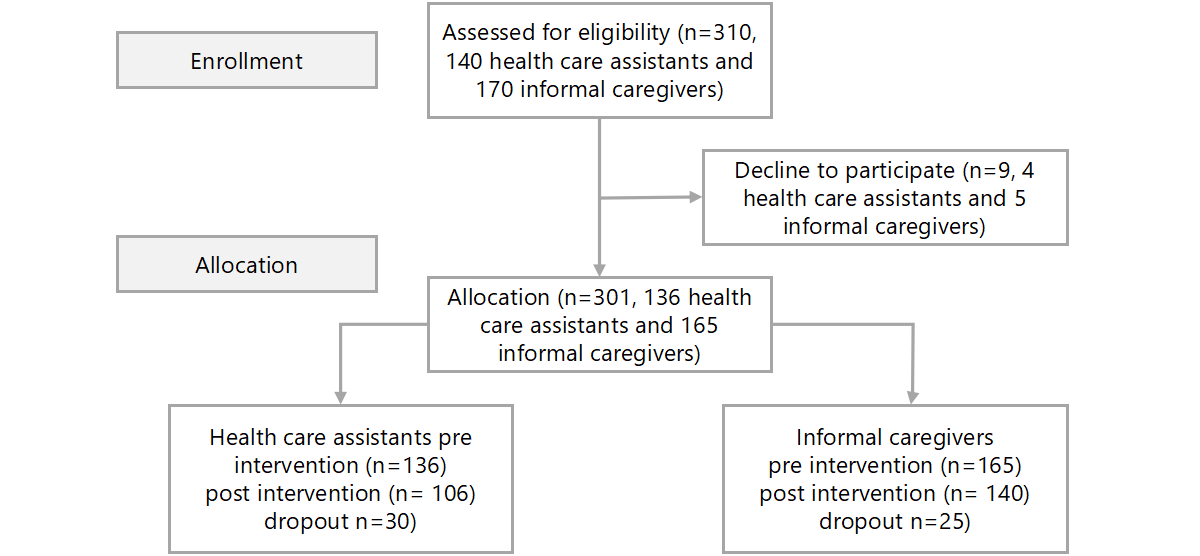
Flow diagram of the progress through the phases of the study. This figure was adapted from Hopewell et al [27], which is published under Creative Commons Attribution 4.0 International License [CC-BY 28]

In total, preassessment data were available for 136 HCAs and 165 ICs (N=301). Of these, 106 HCAs and 140 ICs completed the posttraining assessment (N=246), an attrition of 18.3% (55/301) (Figure 2 [27]). The Little MCAR test indicated data were consistent with MCAR (χ²=8.79, *P*=.36). As prespecified, we further excluded post records that did not meet analytic quality criteria. Reasons for attrition included missing/unusable video recordings (n=21), lack of availability (n=29), and discomfort using VR technology (n=5), resulting in a final sample of 215 participants, which included 94 HCAs and 121 ICs. Overall, the reduction from pre to the analytic data set was 28.6% (86/301).

The mean age of the HCAs was 34.4 (SD 10.2) years, while ICs had a mean age of 47.5 (SD 17.7) years. HCAs operated at a competency level suited to protocol-driven, supervised tasks that relied on basic literacy, communication, and manual skills. Most ICs had completed primary schooling. Table 1 shows the participants’ demographic characteristics.

**Table 1:** Sample characteristics.

Demographic variables		Health care assistants (n=94), n (%)		Informal caregivers (n=121), n (%)
**Gender**				
	Female	72 (76.6)	83 (68.6)	
	Male	22 (23.4)	38 (31.4)	
Age (years), mean (SD)		34.4 (10.2)		47.5 (17.7)

### Rater Concordance

The interrater reliability for error coding was perfect following consensus calibration, with Cohen κ=1, supporting the consistency of rubric-based ratings.

### Correct Execution of Hand Hygiene

Before the intervention, only 26.6% (25/94; 95% CI 18.7-36.3) of HCAs and 9.9% (12/121; 95% CI 5.8-16.5) of ICs performed correct hand hygiene according to guidelines. After the VR training, adherence improved significantly, with 97.9% (92/94; 95% CI 92.6-99.4) of HCAs and 95.9% (116/121; 95% CI 90.7-98.2) of ICs achieving correct execution (*P<*.001 for both HCAs and ICs). Among HCAs, the odds of performing hand hygiene correctly after the intervention were 34.5 times higher than before (95% CI 8.46-140.72), while among ICs, the odds were 21.8 times higher (95% CI 8.90-53.43). Table 2 shows a comparison of hand hygiene performance before and after the intervention.

**Table 2:** Comparison of hand hygiene performance before and after the intervention

Criteria	Health care assistants (n=94)				Informal caregivers (n=121)		
	Pre, n (%)	Post, n (%)	*P* value^a^	Pre, n (%)		Post, n (%)	*P* value
			<.001				<.001
Performed hand hygiene correctly	25 (26.6)	92 (97.9)		12 (9.9)		116 (95.9)	
Did not perform hand hygiene correctly	69 (73.4)	2 (2.1)		109 (90.1)		5 (4.1)	

^a^McNemar test.

### Time Spent on Hand Hygiene

The mean duration of hand hygiene increased from 37.1 (SD 16) seconds (pre) to 46.6 (SD 6.7) seconds (post) (*P*<.001) among HCAs and from 36.5 (SD 12.2) seconds to 48 (SD 6.6) seconds (*P*<.001) among ICs.

### Errors in Hand Hygiene Technique

The most common errors committed by HCAs before training included insufficient coverage of fingertips (44/94, 46.8%; 95% CI 37-56.8), omission of thumb cleaning (32/94, 34%; 95% CI 25.3-44.1), and incomplete nail cleaning by rubbing against the palm (28/94, 29.8%; 95% CI 21.5-39.7). After the intervention, these errors significantly decreased, with all errors dropping to near 0 (*P*<.001 in all cases) (Table 3).

**Table 3:** Types of hand hygiene errors before and after training.

Hand hygiene steps	Health care assistants (n=94)			Informal caregivers (n=121)		
	Pre, n (%)	Post, n (%)	*P* value^a^	Pre, n (%)	Post, n (%)	*P* value
The participant turns on the water tap and wets his/her hands	3 (3.2)	1 (1.1)	0.37	7 (5.8)	0 (0)	.01
Soap is applied	1 (1.1)	1 (1.1)	>.99	16 (13.2)	0 (0)	<.001
The participant rubs his/her palms together	1 (1.1)	0 (0)	>.99	1 (0.8)	1 (0.8)	>.99
Then the participant rubs the top of both hands	5 (5.3)	0 (0)	.06	19 (15.7)	1 (0.8)	<.001
Soap is applied between the fingers of both hands.	15 (16)	0 (0)	<.001	35 (28.9)	1 (0.8)	<.001
The participant rubs his/her hands together, cleaning the inside of their fingers, forming a C shape	44 (46.8)	0 (0)	<.001	83 (68.6)	2 (1.7)	<.001
Nails are cleaned by rubbing them against the palm of the opposite hand	28 (29.8)	0 (0)	<.001	82 (67.8)	1 (0.8)	<.001
The participant washes his/her thumb	32 (34)	0 (0)	<.001	76 (62.8)	1 (0.8)	<.001

^a^Fisher exact test (2-sided).

Similarly, the most common errors among ICs before training were insufficient coverage of fingertips (83/121, 68.6%; 95% CI 60-76.2), incomplete nail cleaning by rubbing against the palm (82/121, 67.8%; 95% CI 59-75.4), and omission of thumb cleaning (76/121, 62.8%; 95% CI: 53.9-70.9). After the intervention, these errors also significantly decreased, with all errors dropping to near 0 (*P*<.001 in all cases).

### Knowledge of Hand Hygiene Practices

The participants’ knowledge significantly improved following the intervention. In the 5-question multiple-choice questionnaire, the mean correct score among HCAs increased from 4.50 (SD 0.52) in the pre phase to 4.60 (SD 0.49) in the post phase, showing a significant increase in correct answers (*P*<.02). ICs showed a similar trend, improving from 4.5 (SD 0.68) to 4.73 (SD 0.5); once again, a significant increase in correct responses was observed in this group (*P*<.001) (Table 4).

**Table 4:** Distribution of total scores on the knowledge questionnaire before and after the intervention

Scores	Health care assistants (n=94)				Informal caregivers (n=121)		
	Pre, n (%)	Post, n (%)	*P* value^a^	Pre, n (%)		Post, n (%)	*P* value
Overall			.02				<.001
1 point	0 (0)	0 (0)		0 (0)		0 (0)	
2 points	0 (0)	0 (0)		1 (0.8)		0 (0)	
3 points	1 (1.1)	0 (0)		10 (8.3)		3 (2.5)	
4 points	45 (47.9)	38 (40.4)		37 (30.6)		27 (22.3)	
5 points	48 (51.1)	56 (59.6)		73 (60.3)		91 (75.2)	

^a^Wilcoxon signed-rank test for paired ordinal totals within each group

### Acceptability and Perception of the VR Training

Most participants reported a positive experience with the VR training. A total of 76.6% (72/94; 95% CI 67.1-84) of HCAs and 90.1% (109/121; 95% CI 83.5-94.2) of ICs found the VR training to be “very useful” or “extremely useful” in improving their hand hygiene practices. However, 7% (15/215; 95% CI 4.3-11.2) of ICs expressed initial difficulties with motion tracking, requiring additional guidance (Table 5). No adverse events (eg, motion sickness, visual fatigue) were reported.

**Table 5:** Usefulness rating of the VRa training (postsession).

Ratings	Health care assistants (n=94), n (%)	Informal caregivers (n=121), n (%)	*P* value^b^
Overall			<.001
Totally useless	17 (18.1)	1 (0.8)	
Slightly useful	0 (0)	0 (0)	
Moderately useful	5 (5.3)	11 (9.1)	
Very useful	36 (38.3)	71 (58.7)	
Extremely useful	36 (38.3)	38 (31.4)	

^a^VR: virtual reality.

^b^Fisher exact test (2-sided) comparing distributions between groups

### Robustness of Results

The LMM with repeated measures confirmed the effectiveness of the intervention over time. Specifically, HCAs committed 1.35 fewer errors post intervention (β=–1.351, *P*<.001), while ICs showed an even larger reduction of 2.58 errors on average (β=–2.579, *P*<.001) (Table 6).

**Table 6:** Linear mixed-effects model for observed errors in health care assistants and informal caregivers.

Fixed effects			β Estimate		Standard error		*t* value (df)		*P* value
**Health care assistants**									
	Intercept	1.372		0.09		15.7 (185.62)		<.001	
	Phase (post)	–1.351		0.12		-11.1 (93.06)		<.001	
**Informal caregivers**									
	Intercept	2.636		0.10		26 (239.62)		<.001	
	Phase (post)	–2.579		0.14		–18.2 (118.47)		<.001	

## Discussion

### Main findings

A single, brief VR-based training produced large, immediate improvements in correct hand hygiene performance in both HCAs and ICs, at the upper end of effects typically seen with WHO-aligned programs using conventional or other active learning formats. Gains coincided with more guideline-concordant timing, disappearance of common execution errors, and high acceptability, suggesting that immersive, feedback-rich practice compresses the skills-acquisition curve. Knowledge increased only modestly, consistent with ceiling effects, indicating that the main value of VR here is behavioral execution rather than declarative knowledge. Taken together, these results support VR as a scalable formative component within multimodal hand hygiene bundles.

### Effectiveness of the Training

The marked improvement in hand hygiene performance observed in this study highlights the potential of VR to overcome common barriers to effective training, including passive learning and lack of real-time feedback. Prior research has demonstrated that immersive technologies, such as VR, can significantly enhance both the technical accuracy and retention of hand hygiene skills [29]. For example, VR training effectively reinforced the WHO Five Moments for Hand Hygiene framework among workers, a critical element for reducing HAIs in clinical practice. Consistent with these findings, this study observed a significant reduction in common errors, such as insufficient fingertip coverage and incomplete nail cleaning, which dropped dramatically. This result supports the hypothesis that active learning based on VR can improve adherence to standardized procedures, even in challenging settings like home care, where ICs often lack formal training [30]. The results also indicate greater adherence to the recommended hand hygiene duration, further supporting the effectiveness of the VR-based intervention.

Throughout the training sessions, no participants reported adverse effects commonly associated with VR, such as motion sickness, visual fatigue, or general discomfort, suggesting good tolerability of the intervention.

Notably, the high effectiveness of the intervention was achieved with a very short training time—only 15 minutes in the longest cases—highlighting the efficiency of the VR-based approach. This minimal time investment, combined with the observed performance improvements, suggests that immersive training not only enhances skill acquisition but does so in a highly time-effective manner. The ability to obtain such substantial gains in technique and adherence in a limited timeframe reinforces the practicality of implementing VR training in busy clinical settings or resource-constrained environments. Additionally, the immersive and interactive nature of VR contributes to increased learner motivation and engagement, offering added value compared to traditional training methods. These advantages position VR as a promising tool for efficient, scalable, and impactful hand hygiene education.

### Feasibility of the Training

Most people—both health care professionals and others—are generally enthusiastic about using new technologies, which creates a motivational gradient that can contribute to positive outcomes [31]. This heightened engagement is particularly beneficial for home care workers [32]. In our study, the technology helped attract participants; however, withdrawals mainly reflected competing caregiving duties at the workplace or at home. Therefore, scheduling in future training editions should accommodate these constraints, especially when multiple sessions are planned over time.

The curriculum aligned with WHO’s recommendations for hand hygiene, which enhanced acceptability and ensured consistency with institutional policies and protocols, mitigating what might otherwise have been a barrier to adoption. In addition, the VR package combined narrated audio guidance with embedded online video, a multimodal design associated with higher engagement and improved skill acquisition, as has been shown in other studies [33].

This experience highlights that individuals with no prior exposure to VR can rapidly adapt to the tool and improve adherence to correct hand hygiene. The learning curve was brief: a few minutes of onboarding to the virtual environment were sufficient—an important consideration for scheduling training sessions and estimating costs. The program mirrored approaches used in other contexts [34], incorporating a rubric-based evaluative component that delivers rapid, actionable feedback on performance within VR.

Training sessions were deliberately brief, enabling high-throughput delivery and broad reach within routine schedules in line with findings in other studies [33,35]. The format was engaging, sustaining attention without increasing time demands. Participant safety safeguards were implemented without difficulty, supporting smooth delivery across sessions.

In this study, a majority of HCAs and practically all ICs rated the VR training as “very useful” or “extremely useful” for improving their hand hygiene practices, reflecting high user satisfaction. This aligns with previous studies suggesting that health care professionals generally prefer VR-based training over traditional methods due to its interactive nature and realistic simulations [29]. The high acceptability observed here may also be attributed to the ability of VR to provide immediate postattempt corrective feedback and personalized learning experiences, which are known to enhance motivation and engagement [36]. However, it is essential to note that a few participants reported discomfort with the technology, highlighting the need for ongoing user support and ergonomic design improvements to reduce dropout rates.

Moreover, the high acceptance rates observed in this study further underscore the advantages of VR as a training tool, while also fostering greater motivation. The participants reported that this blended approach not only facilitated skill acquisition but also significantly enhanced motivation and engagement, critical factors for sustaining long-term behavior change. This aligns with prior research showing that multisensory, interactive learning environments can improve both knowledge retention and practical skill application [37].

### Practical Implications

Although this study was not specifically designed to measure adherence levels to proper hand hygiene practices, the preintervention phase data reveal that only 1 in 10 ICs—who can be considered part of the general population—performed adequate hand hygiene. This finding underscores the notion that poor hand hygiene in community settings represents a significant public health challenge that warrants greater attention.

Previous studies, such as those by Omori et al [38], have noted that VR training can be resource-intensive, requiring considerable investment in hardware, software, and content development. This was also the case in our study, where the initial creation of immersive training scenarios demanded substantial effort and financial resources. However, our experience suggests that once the VR materials are developed, the ongoing costs of implementation and scaling may be comparable to those of traditional training methods, particularly when considering the potential for broader, repeated use. The instructional design of the VR training draws on the Gagné Nine Events [37], guiding learners through structured stages of attention, demonstration, practice, and feedback.

Moreover, the superior effectiveness observed in our study, with near-universal adherence to correct hand hygiene technique after training, suggests that the higher initial costs may be justified by the significant improvements in performance. This balance between upfront investment and long-term impact should be carefully considered in future economic evaluations, as the benefits of reduced HAIs and improved patient safety could offset the initial financial burden. Nonetheless, further studies are needed to comprehensively assess the cost-effectiveness of VR training in various health care contexts, including both direct costs and potential downstream savings from reduced infection rates.

The implementation of VR training programs for ICs could bridge the existing educational gaps, ensuring that these individuals are equipped with the necessary skills to perform effective hand hygiene. Such interventions have the potential to reduce the incidence of HAIs, thereby improving patient outcomes and overall health care quality. The program components were intentionally developed to minimize costs related to equipment and personnel, thereby enhancing their feasibility and scalability across different settings and regions. However, future implementations should consider strategies to ensure equitable access to VR training, particularly in low-resource settings where digital literacy or infrastructure may be limited.

Given the promising results associated with VR training modalities, further research is warranted to explore their efficacy and feasibility among IC populations. Investigating the impact of these technologies on hand hygiene practices could inform the development of targeted educational interventions, ultimately contributing to enhanced infection control measures within health care settings.

Hand hygiene is a fundamental practice in preventing HAIs; however, adherence to proper techniques is often suboptimal. In this study, three-quarters of nursing assistants with continuous patient contact did not perform adequate hand hygiene. This figure rose to 9 out of 10 among the ICs, who also play a critical role in patient care. However, following a brief VR-based training program, adherence rates improved dramatically, confirming the growing body of evidence supporting these disruptive techniques as an effective tool for skill acquisition and behavior modification in health care [36,38].

Taken together, these findings indicate that implementing brief VR training in real-world settings is feasible, provided three conditions are met: (1) flexible scheduling for ICs, including micro-sessions aligned with visiting hours or respite periods, complemented by walk-in availability; (2) protected time for HCAs, secured through shift planning and short back up coverage; and (3) robust data-capture safeguards, including facilitator prompts to keep hands within the tracking field, real-time checks of tracking quality, and procedures that minimize missing data to preserve complete case capture for outcome evaluation.

### Limitations

Despite these promising results, this study has several limitations. First, this study did not include a control group, which limits our ability to compare the intervention against traditional training methods. However, given the well-established effectiveness of hand hygiene in preventing infections, this study focused specifically on assessing the feasibility and performance outcomes of a VR-based training model.

Selection bias is possible due to the snowball sampling approach, and the Hawthorne effect cannot be ruled out because participants knew they were being observed. In addition, secondary outcomes did not inform the sample size calculation and may be underpowered. Generalizability is also constrained by role heterogeneity; the HCA profile varies across facilities, though the results suggest that personnel with limited prior training can achieve good immediate performance under this model. Given the critical role of ICs in infection control in home settings, future studies should evaluate long-term effectiveness in that population, ideally incorporating guidance elements to enhance realism and task fidelity [32].

When interpreting these estimates, note that the McNemar conditional odds ratio depends only on discordant pairs and becomes unstable when one discordant cell is rare. In our data, very few participants worsened after training, creating an extreme imbalance and inflating the variance, hence the wide CIs. We therefore emphasize the paired absolute gains (+71.3 percentage points in HCAs; +86 percentage points in ICs) as a more stable summary of the immediate effect.

Although our data suggest that VR-based training is effective and offers a steep learning gradient relative to the time invested, we did not compare it against traditional methods. A head-to-head evaluation in future studies is warranted, as it may reveal complementary strengths, identify opportunities to optimize VR systems and interfaces, and refine the instructional design (eg, hybrid VR + conventional components), and test whether gains are more durable over time, as has been suggested [39].

The study focused on proximal performance outcomes and did not measure downstream effects on HAI rates, leaving the broader clinical implications of the intervention uncertain. Previous studies have noted that while VR can significantly enhance technique and compliance, the direct impact on patient outcomes remains less clear [38]. Additionally, this study did not assess long-term retention of skills beyond the intervention, a critical factor for sustained behavior change. Therefore, the durability of the observed gains is unknown.

Finally, the study was not designed to quantify costs or conduct a formal economic evaluation. While descriptive observations suggest that costs were predominantly front-loaded (content authoring, hardware configuration, and staff preparation) and that marginal per-participant costs were low, a prospective cost-effectiveness analysis (for example, microcosting and budget-impact assessment) is needed to determine economic value. Despite these limitations, the findings provide proof-of-concept on feasibility, acceptability, participant safety, and adequacy of the training materials, and they can inform the design of a definitive, controlled effectiveness study.

### Conclusion

Active learning facilitated by VR has been shown to enhance performance and ensure the acquisition of essential hand hygiene skills according to standardized procedures. The synergistic effects of these technologies offer a promising path for more effective infection prevention training, particularly for high-risk groups that have historically received less targeted education. Importantly, the high acceptability and positive user experiences observed in this study suggest that immersive training tools not only enhance skill acquisition but also provide a motivating and engaging learning environment. This motivational factor is critical, as it could drive sustained behavior change and ultimately reduce the risk of HAIs and improve patient safety.

## Appendix

Multimedia Appendix 1 Template for intervention description and replication.

Multimedia Appendix 2 TREND (Transparent Reporting of Evaluations with Nonrandomized Designs) statement.

## Abbreviations

CONSORT-EHEALTH: Consolidated Standards of Reporting Trials of Electronic and Mobile Health Applications and Online TeleHealth
HAI: health care–associated infection
HCA: health care assistant
IC: informal caregiver
LMM: linear mixed-effects model
MCAR: Missing Completely at Random
TIDieR: Template for Intervention Description and Replication
TREND: Transparent Reporting of Evaluations with Nonrandomized Designs
VR: virtual reality

## Data Availability

The data sets generated or analyzed during this study are available from the corresponding author on reasonable request.

## References

[1] Pittet D, Allegranzi B, Boyce J, World Health Organization World Alliance for Patient Safety First Global Patient Safety Challenge Core Group of Experts (2009). The World Health Organization guidelines on hand hygiene in health care and their consensus recommendations. Infect Control Hosp Epidemiol.

[2] Bloomfield SF, Aiello AE, Cookson B, O'Boyle C, Larson EL (2007). The effectiveness of hand hygiene procedures in reducing the risks of infections in home and community settings including handwashing and alcohol-based hand sanitizers. American Journal of Infection Control.

[3] WHO (2021). Global progress report on WASH in health care facilities. World Health Organization.

[4] Armstrong-Novak J, Juan HY, Cooper K, Bailey P (2023). Healthcare personnel hand hygiene compliance: are we there yet?. Curr Infect Dis Rep.

[5] Wintaco LM, Quintero-Lesmes DC, Vargas-Soler JA, Barrera DM, Palacio LN, Granados U, Uribe LG (2024). Analysis of healthcare-associated infections before and during the COVID-19 pandemic in a Colombian hospital. Rev Cuid.

[6] Núñez LPC, Martinez GM, Gallo EG (2025). Analysis of a theory of change to evaluate the Health Care-Associated Infection Prevention Program (HAI) in Colombia. BMC Health Serv Res.

[7] Riveros PE, Zambrano P, Amado P (2012). Compliance with hand hygiene guidelines in the intensive care unit: case of a private hospital. Medicina UPB.

[8] Barahona-Guzmán N, Rodríguez-Calderón ME, Rosenthal VD, Olarte N, Villamil-Gómez W, Rojas C, Rodríguez-Ferrer M, Sarmiento-Villa G, Lagares-Guzmán A, Valderrama A, Menco A, Arrieta P, Dajud-Cassas LE, Mendoza M, Sabogal A, Carvajal Y, Silva E (2014). Impact of the International Nosocomial Infection Control Consortium (INICC) multidimensional hand hygiene approach in three cities of Colombia. Int J Infect Dis.

[9] Musu M, Lai A, Mereu NM, Galletta M, Campagna M, Tidore M, Piazza MF, Spada L, Massidda MV, Colombo S, Mura P, Coppola RC (2017). Assessing hand hygiene compliance among healthcare workers in six Intensive Care Units. J Prev Med Hyg.

[10] Lopez-Quintero C, Freeman P, Neumark Y (2009). Hand washing among school children in Bogotá, Colombia. Am J Public Health.

[11] Erasmus V, Daha TJ, Brug H, Richardus JH, Behrendt MD, Vos MC, van Beeck EF (2010). Systematic review of studies on compliance with hand hygiene guidelines in hospital care. Infect Control Hosp Epidemiol.

[12] Liu K, Zhang W, Li W, Wang T, Zheng Y (2023). Effectiveness of virtual reality in nursing education: a systematic review and meta-analysis. BMC Med Educ.

[13] Song J, Jung S, Cheon H, Kim J, Yang Y, Kim I, Kim GJ (2024). Effect of virtual-reality-based education program for managing behavioral and psychological symptoms of dementia: A randomized controlled trial. Geriatr Nurs.

[14] Morganti F, Gattuso M, Singh Solorzano C, Bonomini C, Rosini S, Ferrari C, Pievani M, Festari C (2024). Virtual reality-based psychoeducation for dementia caregivers: the link between caregivers' characteristics and their sense of presence. Brain Sci.

[15] Huang Y, Ho KHM, Christensen M, Wong DW, Wang S, Su JJ, Zhao IY, Kor PPK, Liu JYW, Cheung JC, Leung AYM, Cheung DSK (2024). Virtual reality-based simulation intervention for enhancing the empathy of informal caregivers of people with dementia: A mixed-methods systematic review. Int J Ment Health Nurs.

[16] Thomas MK, Jarrahi AA, Dennie L, Scott S, Lau T, Johnson A (2024). Virtual reality in cancer care: enhancing knowledge and reducing anxiety about chemotherapy among patients and caregivers. Int J Environ Res Public Health.

[17] Ordu Y, Yılmaz S (2024). The effect of using virtual reality goggles on psychological well-being and care burden of informal caregivers of patients hospitalized in a palliative care clinic. Eur J Oncol Nurs.

[18] Patano A, Alanazi M, Lehto R, Goldstein D, Wyatt G (2024). A nature-immersive virtual reality intervention to support hospice family caregivers: Qualitative findings from a pilot study. Asia Pac J Oncol Nurs.

[19] Kokorelias KM, Chiu M, Paul S, Zhu L, Choudhury N, Craven CG, Dubrowski A, Redublo T, Kapralos B, Smith MSD, Shnall A, Sadavoy J, Burhan A (2024). Use of virtual reality and augmented reality technologies to support resilience and skill-building in caregivers of persons with dementia: a scoping review. Cureus.

[20] Luangasanatip N, Hongsuwan M, Limmathurotsakul D, Lubell Y, Lee AS, Harbarth S, Day NPJ, Graves N, Cooper BS (2015). Comparative efficacy of interventions to promote hand hygiene in hospital: systematic review and network meta-analysis. BMJ.

[21] Ojanperä H, Kanste OI, Syrjala H (2020). Hand-hygiene compliance by hospital staff and incidence of health-care-associated infections, Finland. Bull World Health Organ.

[22] Lin M, Huang M, Lai P (2024). Effect of virtual reality training on clinical skills of nursing students: A systematic review and meta-analysis of randomized controlled trials. Nurse Educ Pract.

[23] Hoffmann TC, Glasziou PP, Boutron I, Milne R, Perera R, Moher D, Altman DG, Barbour V, Macdonald H, Johnston M, Lamb SE, Dixon-Woods M, McCulloch P, Wyatt JC, Chan A, Michie S (2014). Better reporting of interventions: template for intervention description and replication (TIDieR) checklist and guide. BMJ.

[24] Des Jarlais DC, Lyles C, Crepaz N (2004). Improving the reporting quality of nonrandomized evaluations of behavioral and public health interventions: the TREND statement. Am J Public Health.

[25] Eysenbach G, CONSORT- E (2011). CONSORT-EHEALTH: improving and standardizing evaluation reports of web-based and mobile health interventions. J Med Internet Res.

[26] Sahito ZH, Khoso FJ, Phulpoto J (2025). The effectiveness of active learning strategies in enhancing student engagement and academic performance. JSSR.

[27] Hopewell S, Chan A-W, Collins GS, Hróbjartsson A, Moher D, Schulz KF, Tunn R, Aggarwal R, Berkwits M, Berlin JA, Bhandari N, Butcher NJ, Campbell MK, Chidebe RCW, Elbourne D, Farmer A, Fergusson DA, Golub RM, Goodman SN, Hoffmann TC, Ioannidis JPA, Kahan BC, Knowles RL, Lamb SE, Lewis S, Loder E, Offringa M, Ravaud P, Richards DP, Rockhold FW, Schriger DL, Siegfried NL, Staniszewska S, Taylor RS, Thabane L, Torgerson D, Vohra S, White IR, Boutron I (2025). CONSORT 2025 statement: updated guideline for reporting randomised trials. BMJ.

[28] Attribution 4.0 International (CC BY 4.0). Creative Commons.

[29] Eichel VM, Brandt C, Brandt J, Jabs JM, Mutters NT (2022). Is virtual reality suitable for hand hygiene training in health care workers? Evaluating an application for acceptability and effectiveness. Antimicrob Resist Infect Control.

[30] Wang J, Li Q, Cui J, Tu S, Deng Z, Yang R, Wang Y (2023). Effectiveness of virtual reality on the caregiving competence and empathy of caregivers for elderly with chronic diseases: a systematic review and meta-analysis. J Nurs Manag.

[31] Kyaw BM, Saxena N, Posadzki P, Vseteckova J, Nikolaou CK, George PP, Divakar U, Masiello I, Kononowicz AA, Zary N, Tudor Car L (2019). Virtual reality for health professions education: systematic review and meta-analysis by the digital health education collaboration. J Med Internet Res.

[32] Gasteiger N, van der Veer SN, Wilson P, Dowding D (2023). Virtual reality and augmented reality smartphone applications for upskilling care home workers in hand hygiene: a realist multi-site feasibility, usability, acceptability, and efficacy study. J Am Med Inform Assoc.

[33] Kubr J, Lochmannová A, Hořejší P (2024). Immersive virtual reality training in industrial settings: effects on memory retention and learning outcomes. IEEE Access.

[34] Bednár M, Dufek P, Lochmannová A, Simon M, Bures M (2023). Use of VR for education and training of emergency rescue system for crisis situations. https://doi.org/10.1145/3629296.3629318.

[35] Alanazi MO, Patano A, Bente G, Mason A, Goldstein D, Parsnejad S, Wyatt G, Lehto R (2023). Nature-based virtual reality feasibility and acceptability pilot for caregiver respite. Curr Oncol.

[36] McNeill L, Fitch D (2023). Microlearning through the lens of Gagne's Nine Events of Instruction: a qualitative study. TechTrends.

[37] Stuart JP, Gannon PR, Dotto VR, Regina R, Mumma JM (2025). Visualizing the WHO "My Five Moments for Hand Hygiene," framework: A virtual reality training program for improving hand hygiene adherence among nurses. Am J Infect Control.

[38] Omori K, Shigemoto N, Kitagawa H, Nomura T, Kaiki Y, Miyaji K, Akita T, Kobayashi T, Hattori M, Hasunuma N, Tanaka J, Ohge H (2023). Virtual reality as a learning tool for improving infection control procedures. Am J Infect Control.

[39] Lochmannová A (2025). Exploring the role of virtual reality in preparing emergency responders for mass casualty incidents. Isr J Health Policy Res.

