# Supplementary material for: Improving Hand Hygiene Skills Using Virtual Reality: Quasi-Experimental Study
Source: J Med Internet Res. 2025 Oct 7;27:e78882. doi: 10.2196/78882 (PMC12541262; doi:10.2196/78882)
Supplement: Multimedia Appendix 1 [file jmir_v27i1e78882_app1.pdf]

TIDieR Checklist — RealityCare VR Hand-Hygiene Training

Study: Improving Hand Hygiene Skills Using Virtual Reality: A Quasi-Experimental Study (Colombia)
Registration: ClinicalTrials.gov NCT07005544
Reporting guideline: TIDieR (BMJ 2014;348:g1687)

Item	TIDieR component	Details for this study
1	1. Brief name	VR Hand-Hygiene Training
2	2. Why (rationale, theory, or goal of elements essential to the intervention)	To address persistent technique and timing deficits in hand hygiene among healthcare assistants and informal caregivers using active learning with immediate feedback. Alignment with WHO's recomendations was intended to enhance acceptability and policy fit and to standardize behavioural execution for rapid skill acquisition.
3	3. What (materials)	Standalone VR scenario simulating a complete soap-and-water handwashing sequence; narrated audio plus an embedded instructional video (~1:27); performance rubric/checklist aligned with WHO recommendations; standardised onboarding script (headset fit, basic navigation, required gestures); disinfectable headset covers and cleaning supplies; data-capture forms/logs.
4	4. What (procedures)	Baseline PRE performance (as usual); standardized orientation (3–5 min) to minimize motion-tracking issues and ensure smooth onboarding; 1–3 individual VR practice micro-sessions with real-time visual/audio feedback; POST assessment using the same rubric; knowledge questionnaire; device cleaning between users; safety checks and prompts to keep hands within tracking field.
5	5. Who provided (expertise, background)	Trained facilitators (nurses) from participating hospitals, oriented by the technical lead. Roles: deliver orientation and safety checks, provide standardized prompts, monitor tracking quality, and record outcomes.

6	6. How (modes of delivery)	In-person, one-to-one delivery in VR with a facilitator present; no group training or remote delivery.
7	7. Where (locations)	Seven private hospitals in Bogotá, Colombia. Sessions conducted in quiet spaces adjacent to clinical areas. Participants included HCAs and informal caregivers voluntary involved.
8	8. When and how much (schedule, intensity, dose)	Orientation 3–5 minutes; up to three 15-minute VR sessions per participant (micro-sessions compatible with routine schedules). The maximum total VR exposure per participant was 45 minutes; actual exposure could be less depending on performance.
9	9. Tailoring (what, why, when, how)	Headset fit and comfort adjusted; pacing adapted to participant speed; repeated prompts as needed; core content and assessment rubric unchanged.
10	10. Modifications (during study)	No protocol modifications after study start.
11	11. How well—planned (fidelity strategies)	Standardized facilitator checklist and scripts; pre-session device calibration; motion-tracking quality checks; data-completeness procedures; predefined prompts to maintain hands in frame.
12	12. How well—actual (adherence/fidelity)	Session logs captured errors and timing; rater agreement procedures implemented; reasons for attrition recorded (e.g., availability, discomfort). See Results for acceptability and completion rates.
